# Biochemical Characterization of a Purified Glycosylated β-Fibrinogenolytic Serine Protease Preparation from *Gloydius ussuriensis* Venom

**DOI:** 10.3390/biology15201892

**Published:** 2026-10-20

**Authors:** Eu-Ri Jo, Jung Eun Park

**Affiliations:** 1 Department of Biomedical Science, College of Natural Sciences, Chosun University, Gwangju 61452, Republic of Korea; 2 Department of Integrative Biological Science & BK21 FOUR Educational Research Group for Age-Associated Disorder Control Technology, Chosun University, Gwangju 61452, Republic of Korea

**Keywords:** snake venom, serine protease, fibrinogenolysis, thermostability, glycoprotein, *Gloydius ussuriensis*, thrombin-like activity

## Abstract

Snake-venom serine proteases can alter hemostasis through diverse effects on fibrinogen and coagulation factors. Here, a heat-resistant protease under the tested conditions, designated TLSP, was purified from *Gloydius ussuriensis* venom by sequential anion-exchange and size-exclusion chromatography and characterized biochemically. TLSP migrated at approximately 35 kDa under reducing conditions and 26 kDa under non-reducing conditions. Periodic acid–Schiff staining and N-glycosidase F treatment indicated N-linked glycosylation. The N-terminal sequence, VVGGDEHNIN, showed high similarity to reported snake-venom thrombin-like serine proteases. TLSP preferentially hydrolyzed Boc-Val-Pro-Arg-*pNA*, with *K*m = 0.3 mM and *k*cat = 8.69 s^−1^, and its activity was strongly inhibited by aprotinin and moderately reduced by PMSF. TLSP preferentially cleaved the β-chain of human fibrinogen but did not induce fibrin-polymer formation or degrade preformed or factor XIIIa-cross-linked fibrin. The optimum pH was approximately 9.0, and 59% of amidolytic activity remained after heat treatment at 100 °C. N-glycosidase F treatment was accompanied by loss of detectable amidolytic activity. These findings characterize TLSP as a glycosylated β-fibrinogenolytic serine protease showing marked heat resistance under the tested conditions, with thrombin-like substrate preference but without thrombin-like fibrin-clotting activity.

## Simple Summary

Snake venoms contain many enzymes that alter blood clotting. In this study, a protease was purified from *Gloydius ussuriensis* venom and examined for its effects on fibrinogen and related coagulation proteins. The enzyme preferentially cleaved the β-chain of fibrinogen but did not convert fibrinogen into a fibrin clot and did not digest preformed fibrin. It remained strongly active after heating and retained approximately 59% of its activity after exposure to 100 °C. The protein was glycosylated, and N-glycosidase F treatment was accompanied by loss of detectable activity toward the preferred chromogenic substrate. These properties distinguish the enzyme from physiological thrombin and support its characterization as a glycosylated, β-fibrinogenolytic serine protease showing marked heat resistance under the tested conditions, together with thrombin-like amidolytic properties. The findings provide a biochemical basis for understanding how snake-venom proteases interact with the coagulation system and provide a foundation for future molecular identification and structure–function studies.

## 1. Introduction

Snake venoms are complex mixtures of biologically active proteins and peptides that can affect neuromuscular function, tissue integrity, vascular permeability, platelet responses, and the hemostatic system [1,2]. Among the enzymatic components, serine proteases and metalloproteases are particularly important because they can modify multiple steps of blood coagulation and fibrinolysis [3,4,5]. Snake-venom serine proteases include thrombin-like enzymes, kinin-releasing enzymes, factor-activating proteases, and other enzymes that act on coagulation-related substrates [3,6,7].

Snake-venom thrombin-like enzymes (SVTLEs) share functional features with thrombin but differ substantially in substrate recognition and biological outcomes [8]. Many SVTLEs preferentially act on one fibrinogen chain and have therefore been described as α- or β-fibrinogenases, whereas thrombin cleaves fibrinogen in a coordinated manner to generate fibrin monomers that polymerize into a clot [8,9]. Several fibrinogen-active SVTLEs have been characterized across viperid venoms, including halystase and contortrixobin, which preferentially target the fibrinogen β-chain [7,10]. These differences in substrate selectivity are relevant to the effects of venom proteases on coagulation and to their potential use as biochemical tools.

Proteases have previously been described from *Gloydius* venoms, including thrombin-like serine proteases [11,12]. In the present study, we biochemically characterized a serine protease, designated TLSP, purified from *Gloydius ussuriensis* venom, historically referred to as *Gloydius halys ussuriensis* in the original source records. Particular emphasis was placed on its purification, N-terminal sequence, glycosylation, chromogenic-substrate specificity, fibrinogenolytic activity, interactions with coagulation-related proteins, inhibitor sensitivity, and pH and thermal stability. The principal objective was to define the biochemical phenotype of TLSP and to distinguish its thrombin-like amidolytic properties from true thrombin-like fibrin-clotting activity.

## 2. Materials and Methods

### 2.1. Materials

Lyophilized crude venom of *Gloydius ussuriensis* (referred to as *Gloydius halys ussuriensis* in the original source records) was obtained from Latoxan (Valence, France). HiPrep 16/10 Q FF, Source 15 Q 4.6/100 PE, Superdex 75 10/300 GL, Mono Q HR 5/5, and PD-10 columns were obtained from Amersham Biotech (Uppsala, Sweden). Boc-Val-Pro-Arg-*pNA* (Boc-VPR-*pNA*) and Boc-Leu-Gly-Arg-*pNA* (Boc-LGR-*pNA*) were obtained from Seikagaku (Tokyo, Japan). S-2222, S-2238, S-2251, S-2288, S-2765, and S-2444 were obtained from Chromogenix (Raleigh, NC, USA). Protease inhibitors and other routine reagents were obtained from Sigma unless otherwise indicated. Human prothrombin was obtained from CalBiochem (San Diego, CA, USA), and human plasminogen and plasmin were obtained from Roche Applied Science (Mannheim, Germany). N-glycosidase F was obtained from New England BioLabs (Ipswich, MA, USA). Human fibrinogen was obtained from Sigma (St. Louis, MO, USA).

### 2.2. Purification of TLSP

Lyophilized crude venom (103.26 mg) was dissolved in 3 mL of 20 mM Tris-HCl (pH 8.0) and centrifuged at 13,000× *g* for 1 min. The supernatant was applied to a HiPrep 16/10 Q FF column equilibrated with the same buffer, and bound proteins were eluted with a 0–0.5 M NaCl gradient at 2.5 mL/min. Fractions showing amidolytic activity toward Boc-VPR-*pNA* were pooled and concentrated using an Amicon YM-10 membrane (Amicon, Beverly, MA, USA). The concentrate was applied to a Source 15 Q 4.6/100 PE column and eluted with a 0–0.25 M NaCl gradient at 0.7 mL/min. Active fractions were then separated on a Superdex 75 10/300 GL column (Cytiva, Uppsala, Sweden) in 20 mM Tris-HCl (pH 8.0) containing 200 mM NaCl at 0.5 mL/min. The active pool was applied to a Mono Q HR 5/5 column (Cytiva, Uppsala, Sweden) and eluted with a 0–0.2 M NaCl gradient at 0.7 mL/min; the major active fraction was subjected to a second Mono Q step. The final active fraction was used as purified TLSP. Protein concentration was determined using Bradford reagent.

### 2.3. SDS-PAGE and Glycoprotein Analysis

Protein samples were analyzed using 8%, 10%, or 12% SDS-polyacrylamide gels under reducing or non-reducing conditions as appropriate. Protein bands were visualized with Coomassie brilliant blue. Glycoprotein staining was performed with periodic acid–Schiff (PAS) reagent. For deglycosylation, TLSP (2 µg) was treated with N-glycosidase F at 37 °C for 16 h according to the manufacturer’s procedure. Reaction products were analyzed by SDS-PAGE. The completeness of deglycosylation was not independently assessed.

### 2.4. N-Terminal Amino Acid Sequencing

Purified TLSP was separated by 12% SDS-PAGE and transferred to a PVDF membrane using 10 mM 3-(cyclohexylamino)-1-propanesulfonic acid buffer (pH 11.0) containing 10% methanol. After Coomassie staining and destaining, the target band was excised. N-terminal amino acid sequencing was performed at the Korea Basic Science Institute (Seoul, Republic of Korea).

### 2.5. Amidolytic Activity and Enzyme Kinetics 

Amidolytic activity was measured in a 100 µL reaction containing 1 µg TLSP, 20 mM Tris-HCl (pH 8.0), 0.2 M NaCl, 0.1 mg/mL bovine serum albumin, and 0.4 mM chromogenic substrate. Boc-VPR-*pNA* and S-2238 were used as thrombin-directed substrates; Boc-LGR-*pNA*, S-2222, and S-2765 as factor Xa-directed substrates; S-2251 as a chromogenic substrate for plasmin; S-2288 as a t-PA/broad serine-protease substrate; and S-2444 as a urokinase-directed substrate. Reactions were incubated at 37 °C for 20 min, and substrate hydrolysis was monitored at 405 nm. Kinetic measurements were performed in two independent experiments over a Boc-VPR-*pNA* concentration range of 0–0.6 mM using 0.5 µg TLSP. *K*m and *k*cat were estimated by fitting the initial-velocity data to the Michaelis–Menten equation.

### 2.6. Fibrinogenolytic and Fibrin-Clotting Assays

For the concentration-dependent fibrinogenolysis assay, human fibrinogen (200 µg) was incubated with 1, 3, 5, or 10 µg TLSP in 200 µL of 20 mM Tris-HCl (pH 8.0) at 37 °C for 2 h, followed by SDS-PAGE. For the time-course experiment, TLSP (12.5 µg) was incubated with fibrinogen (250 µg) in 250 µL at 37 °C, and aliquots were collected from 0.5 to 24 h. The concentration-dependent assay used enzyme-to-substrate mass ratios of 1:200 to 1:20, whereas the time-course experiment used a ratio of 1:20. Fibrin-clot formation was assessed by monitoring turbidity at 350 nm after incubation of fibrinogen with TLSP; thrombin served as a positive control.

### 2.7. Fibrinolytic Activity and Cross-Linked Fibrin

Fibrin polymer was generated by incubating 90 µL of fibrinogen (1 mg/mL) with 10 µL of thrombin (10 U/mL) for 1 h at room temperature. TLSP (5 µg) or plasmin (1 µg) was then added, and changes in turbidity at 350 nm were monitored after 2, 4, and 16 h. Fibrinolysis was also assessed using fibrin plates. To evaluate cross-linked fibrin degradation, fibrin was polymerized in the presence of thrombin, factor XIIIa, and CaCl_2_ and then incubated with TLSP; products were analyzed by SDS-PAGE.

### 2.8. Cleavage of Prothrombin and Plasminogen

TLSP (3 µg) was incubated with prothrombin or plasminogen (40 µg) in 80 µL of 20 mM Tris-HCl (pH 8.0) at 37 °C. Aliquots were collected after 4, 8, and 24 h and analyzed by SDS-PAGE. To determine whether TLSP-generated prothrombin fragments possessed thrombin activity, prothrombin was incubated with or without TLSP for 24 h, followed by addition of fibrinogen. Fibrin formation was monitored by turbidity at 350 nm, with thrombin as a positive control.

### 2.9. Effects of Protease Inhibitors and Divalent Cations

TLSP (1 µg) was pre-incubated for 10 min at 37 °C with EGTA, PMSF, aprotinin, bestatin, 1,10-phenanthroline, EDTA, TPCK, TLCK, or DFP (1 mM each), or with β-mercaptoethanol or DTT (500 mM), before residual activity was measured using Boc-VPR-*pNA*. In parallel, the effects of Ni^2+^, Zn^2+^, Mg^2+^, Mn^2+^, Cu^2+^, and Ca^2+^ were tested at a final concentration of 1 mM. Activities were expressed relative to an untreated control.

### 2.10. Effects of pH and Temperature

The pH profile of TLSP was measured at pH 4.0–12.0 using 50 mM sodium acetate (pH 4.0 and 5.0), 50 mM sodium phosphate (pH 6.0 and 7.0), 50 mM Tris-HCl (pH 8.0 and 9.0), 50 mM glycine-NaOH (pH 10.0 and 11.0), and 50 mM sodium carbonate (pH 12.0). For thermal-stability testing, TLSP was pre-incubated from 4 to 100 °C for 30 min in Tris-HCl buffer and then assayed with Boc-VPR-*pNA*. Preservation of fibrinogenolytic activity after heat treatment was evaluated by pre-incubating TLSP at 37 °C or 100 °C for 30 min and subsequently incubating it with fibrinogen for 1 or 2 h at 37 °C; the corresponding figure legend reports 2 µg TLSP for this experiment.

### 2.11. Data Presentation

Quantitative assays were generally performed in duplicate unless otherwise specified. Kinetic measurements were performed in two independent experiments (*n* = 2). Results are presented as mean ± SD where applicable. Electrophoretic images are shown as representative results. No inferential statistical analyses were performed; therefore, the quantitative data are interpreted descriptively.

## 3. Results

### 3.1. Purification and Glycosylation of TLSP

**Figure 1:**
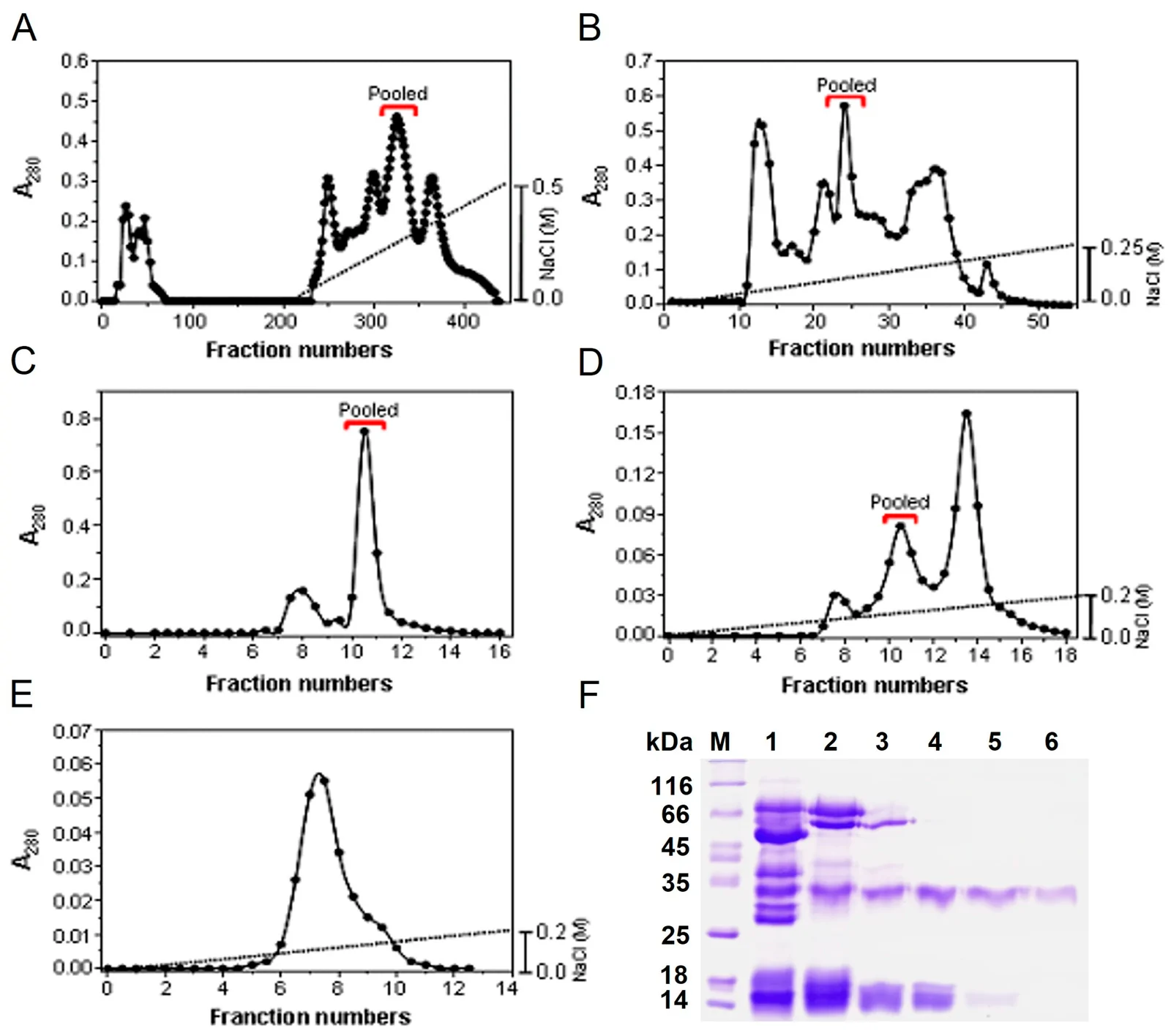
**Purification of TLSP from *Gloydius ussuriensis* venom.** Sequential chromatographic purification using (**A**) HiPrep 16/10 Q FF, (**B**) Source 15 Q, (**C**) Superdex 75, and (**D**,**E**) two Mono Q steps. Activity-containing fractions were tracked using Boc-VPR-*pNA*. (**F**) SDS-PAGE of proteins obtained during purification. Lane M, molecular-weight markers; lane 1, crude venom; lane 2, HiPrep Q; lane 3, Source Q; lane 4, Superdex 75; lanes 5 and 6, first and second Mono Q fractions.

Boc-VPR-*pNA*-hydrolyzing activity was tracked through five chromatographic steps: HiPrep 16/10 Q FF, Source 15 Q, Superdex 75, and two sequential Mono Q separations (Figure 1A–E). SDS-PAGE showed progressive enrichment of a major protein band during purification (Figure 1F). Starting from 103.26 mg of crude-venom protein and 6195 U of total activity, the final preparation contained 0.14 mg of protein and 140 U of activity, corresponding to a specific activity of 1000 U/mg and a yield of 2.3% (Table 1).

**Figure 2:**
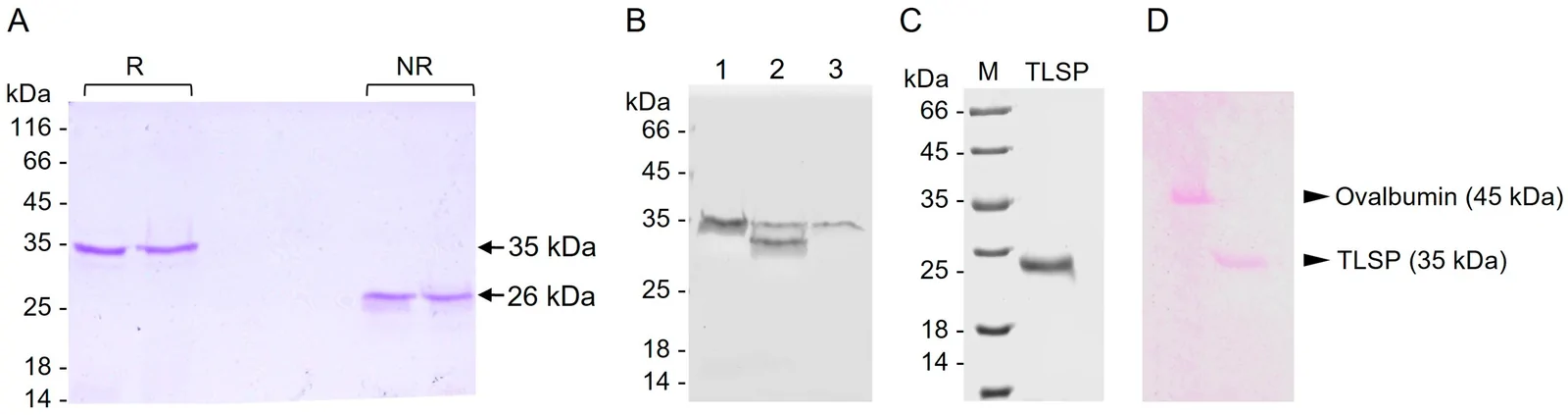
**Electrophoretic and glycoprotein characterization of purified TLSP.** (**A**) SDS-PAGE analysis of purified TLSP under reducing (R) and non-reducing (NR) conditions. The apparent molecular masses of TLSP were approximately 35 kDa and 26 kDa under reducing and non-reducing conditions, respectively. (**B**) SDS-PAGE analysis of TLSP following treatment with N-glycosidase F. Lane 1, untreated TLSP; lane 2, N-glycosidase F-treated TLSP; lane 3, N-glycosidase F alone. N-glycosidase F treatment resulted in a decrease in the apparent molecular mass of TLSP. (**C**) Coomassie brilliant blue staining of purified TLSP. Lane M, molecular mass markers; TLSP, purified TLSP. (**D**) Periodic acid–Schiff (PAS) staining of purified TLSP. Ovalbumin (45 kDa) was used as a glycoprotein-positive control. PAS staining of TLSP supported the presence of covalently linked carbohydrate moieties.

Purified TLSP migrated at approximately 35 kDa under reducing conditions and 26 kDa under non-reducing conditions (Figure 2A). Coomassie brilliant blue staining showed a major purified TLSP band, while PAS staining supported the presence of covalently linked carbohydrate moieties (Figure 2C,D). Treatment with N-glycosidase F reduced the apparent molecular mass of TLSP under reducing conditions from approximately 35 kDa to 33 kDa, corresponding to an apparent shift of approximately 2 kDa (Figure 2B). An N-glycosidase F-only control was included in the electrophoretic analysis. Together, these findings support the presence of N-linked glycosylation in TLSP.

### 3.2. Kinetic Properties, N-Terminal Sequence, and Chromogenic-Substrate Specificity

Using Boc-VPR-pNA as substrate, TLSP had a *K*m of 0.3 mM and a *k*cat of 8.69 s^−1^, corresponding to a catalytic efficiency (*k*cat*/K*m) of 28.8 mM^−1^ s^−1^ (Table 2 and Supplementary Figure S1). The N-terminal sequence of TLSP (VVGGDEHNIN) showed 90% identity with VLβF, 80% with calobin, 90% with contortrixobin, and 30% with human α-thrombin (Table 3). These sequence similarities support classification of TLSP within the snake-venom serine-protease family but do not establish its exact molecular identity.

Among the chromogenic substrates tested, Boc-VPR-*pNA* produced the highest relative activity and was set to 100%. Boc-LGR-*pNA* and S-2288 also showed substantial hydrolysis, at 85% and 81% relative activity, respectively. Activities toward S-2222, S-2238, S-2251, S-2765, and S-2444 were lower (Table 4). Thus, TLSP displayed a broad amidolytic profile with a preference for Boc-VPR-*pNA* rather than strict thrombin-like substrate specificity.

### 3.3. TLSP Preferentially Cleaves the Fibrinogen β-Chain but Does Not Form Fibrin Polymer

**Figure 3:**
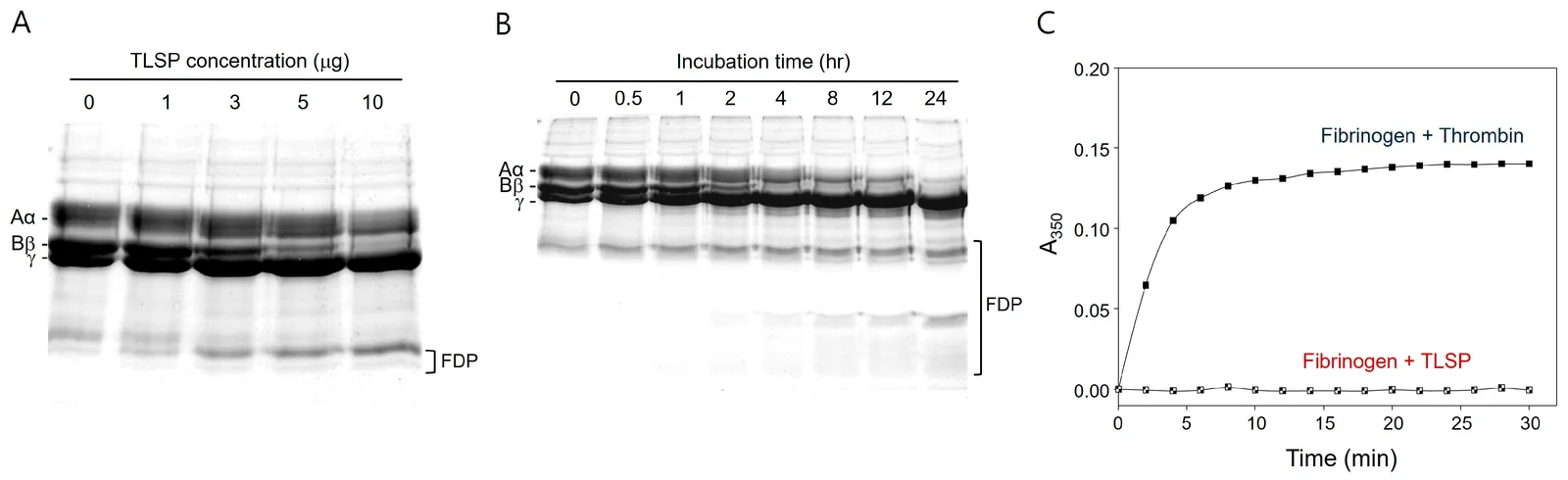
**Fibrinogenolytic activity of TLSP.** (**A**) Human fibrinogen (200 µg) was incubated with increasing amounts of TLSP (1, 3, 5, or 10 µg) for 2 h at 37 °C and analyzed by SDS-PAGE. (**B**) Time-dependent degradation of human fibrinogen by TLSP. (**C**) Fibrin-polymer formation assessed by turbidity at 350 nm. The original protein gel images are provided in Supplementary File S1. TLSP did not induce the turbidity increase observed with thrombin. FDP, fibrinogen degradation products; TLSP, thermostable serine protease.

In the concentration-dependent experiment, TLSP cleaved the fibrinogen β-chain strongly within 2 h at 37 °C, whereas the α-chain was less susceptible (Figure 3A). In a separate time-course experiment performed with a different enzyme-to-substrate ratio, complete β-chain degradation was evident by approximately 4 h, while marked α-chain degradation became apparent only after prolonged incubation, approximately 12 h or longer (Figure 3B). The SDS-PAGE data qualitatively showed progressive degradation of the fibrinogen β-chain, followed by α-chain degradation after prolonged incubation. Despite its fibrinogenolytic activity, TLSP did not increase fibrin turbidity, whereas thrombin rapidly induced fibrin-polymer formation (Figure 3C). These observations indicate preferential β-fibrinogenolysis without fibrin-clotting activity.

### 3.4. Effects on Fibrin, Cross-Linked Fibrin, Plasminogen, and Prothrombin

**Figure 4:**
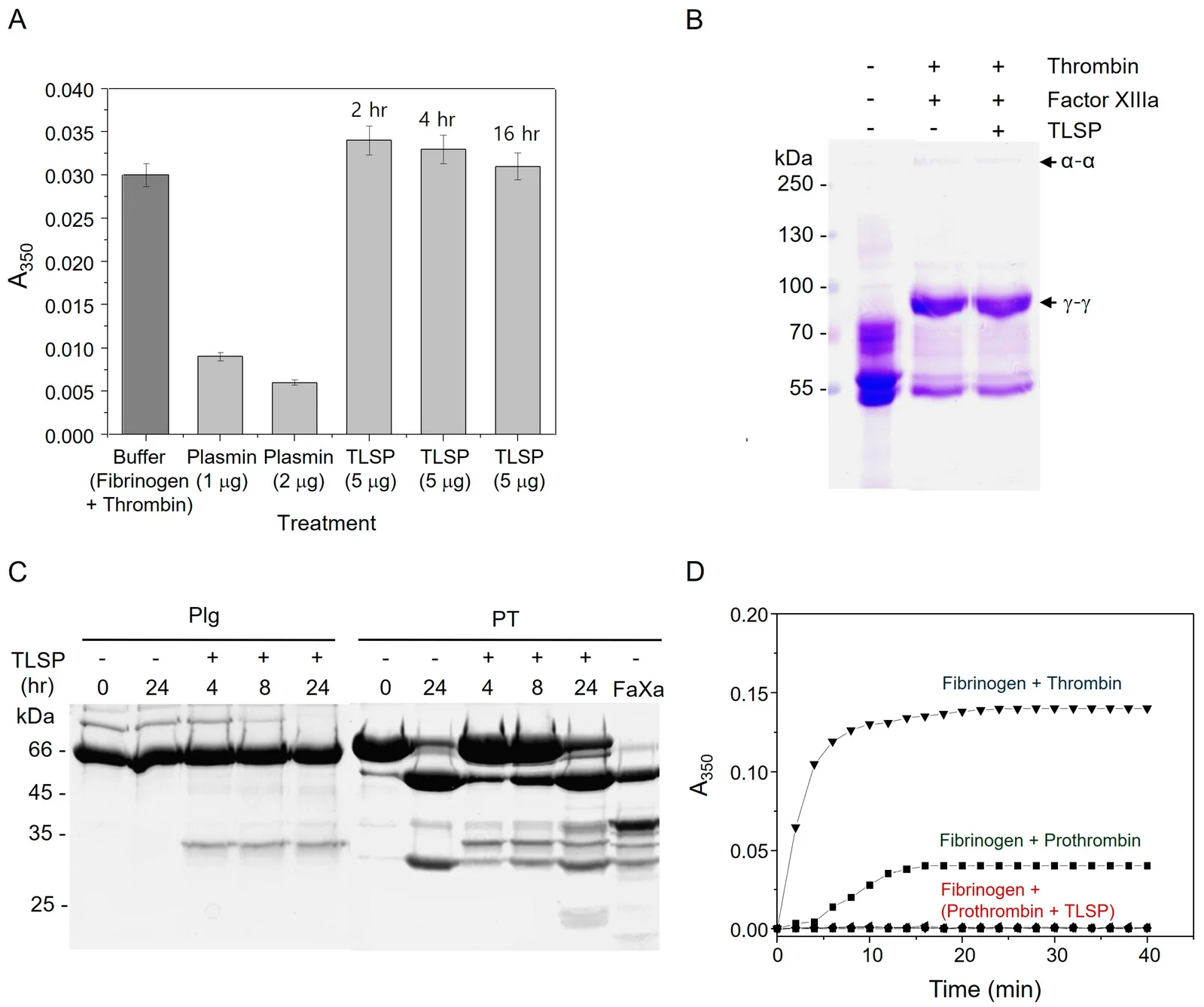
**Effects of TLSP on coagulation-related substrates.** (**A**) Fibrinolytic activity measured by changes in fibrin-polymer turbidity; plasmin served as a positive control. (**B**) SDS-PAGE analysis of factor XIIIa-cross-linked fibrin after TLSP treatment. (**C**) Cleavage of plasminogen and prothrombin by TLSP. (**D**) Assessment of fibrin formation by TLSP-generated prothrombin products. TLSP cleaved both proteins during prolonged incubation, but prothrombin cleavage products did not display thrombin activity. The original protein gel images are provided in Supplementary File S1. Plg, plasminogen; PT, prothrombin; TLSP, thermostable serine protease.

TLSP did not show plasmin-like fibrinolytic activity against preformed fibrin polymer in the turbidity assay, in contrast to plasmin (Figure 4A). It also failed to cleave factor XIIIa-cross-linked fibrin under the conditions tested (Figure 4B). TLSP nevertheless cleaved plasminogen and prothrombin during prolonged incubation. The prothrombin cleavage products did not induce fibrin formation, indicating that TLSP-mediated proteolysis did not generate thrombin activity (Figure 4D). Together, these findings further distinguish the biochemical phenotype of TLSP from both plasmin and physiological thrombin.

### 3.5. Effects of Protease Inhibitors and Divalent Cations

TLSP activity was strongly inhibited by aprotinin and moderately reduced by PMSF. β-Mercaptoethanol and DTT, tested at 500 mM, reduced the residual activity of TLSP to 31.6% and 15.5%, respectively. In contrast, EGTA, EDTA, 1,10-phenanthroline, bestatin, TPCK, and DFP showed little or no inhibitory effect, whereas TLCK produced only a modest reduction in activity (Table 5). 

Among the divalent cations tested at 1 mM, Ni^2+^, Zn^2+^, and Cu^2+^ markedly inhibited TLSP activity, reducing the residual activity to 9.1%, 1.4%, and 17.5%, respectively. In contrast, Mg^2+^, Mn^2+^, and Ca^2+^ had little effect, with residual activities of 99.3%, 98.5%, and 97.2%, respectively (Table 6).

### 3.6. Alkaline pH Preference, Heat Resistance, and Effect of N-Glycosidase F Treatment

**Figure 5:**
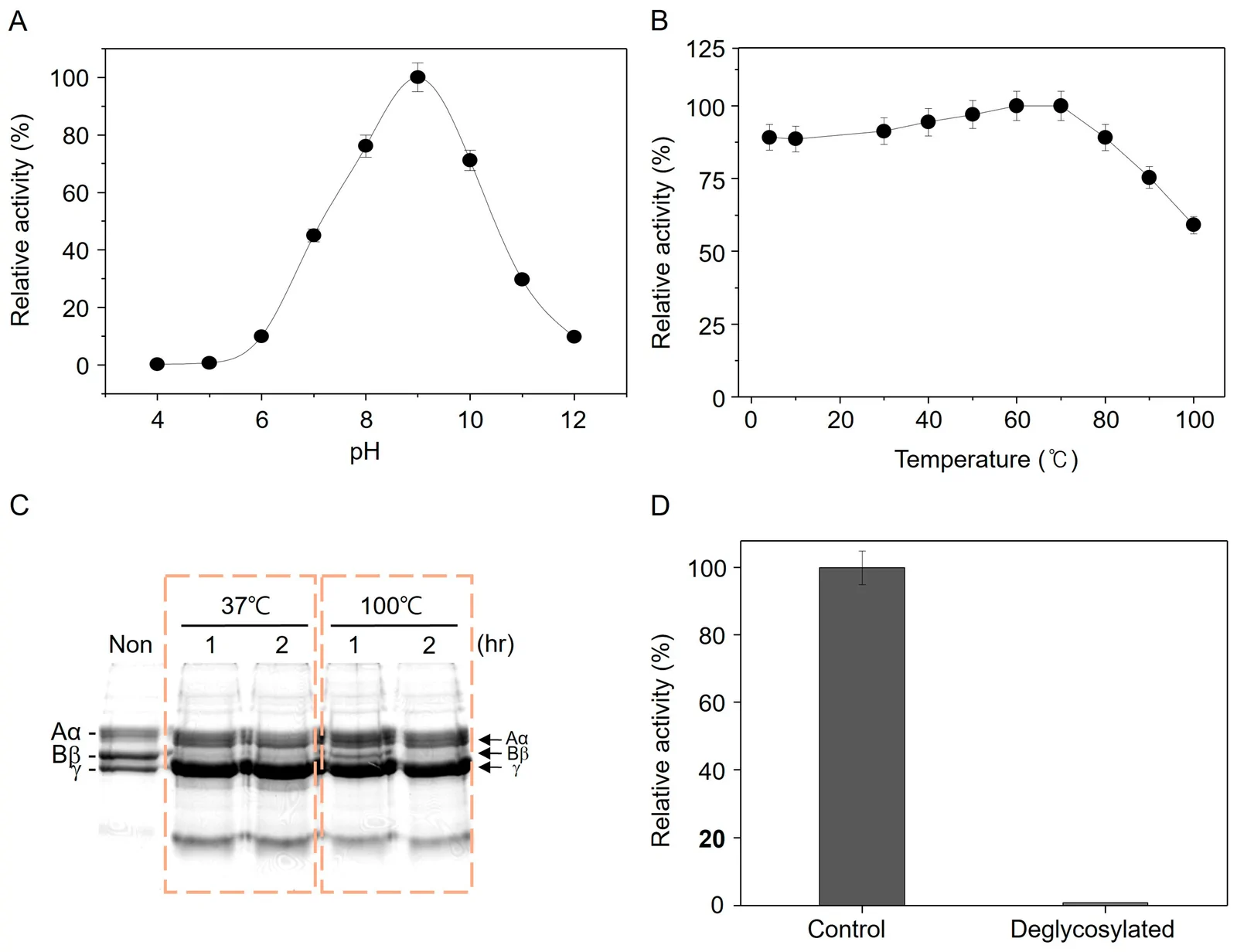
**pH dependence, heat resistance, and effect of N-glycosidase F treatment on TLSP.** (**A**) Relative activity across pH 4–12, with maximal activity near pH 9.0. (**B**) Residual activity after temperature pre-incubation. (**C**) Preservation of fibrinogen β-chain cleavage after exposure to 100 °C. (**D**) Loss of detectable Boc-VPR-*pNA*-hydrolyzing activity after N-glycosidase F treatment. The original protein gel images are provided in Supplementary File S1.

TLSP displayed maximal activity near pH 9.0 and maintained high activity over an alkaline range of approximately pH 8–10 (Figure 5A). After 30 min pre-incubation at increasing temperatures, TLSP retained measurable activity throughout the tested range; notably, approximately 59% of activity remained after exposure to 100 °C (Figure 5B). Enzyme pre-incubated at 100 °C also retained the ability to cleave the fibrinogen β-chain (Figure 5C). N-glycosidase F treatment was accompanied by loss of detectable Boc-VPR-*pNA*-hydrolyzing activity under the conditions tested (Figure 5D). However, this endpoint assay does not establish whether the loss of activity resulted from altered folding, active-site accessibility, stability, or another structural effect.

## 4. Discussion

The biochemical data characterize TLSP as a glycosylated snake-venom serine protease with a distinctive combination of thrombin-like amidolytic preference, preferential β-fibrinogenolysis, absence of fibrin-clotting activity, and marked thermal resistance. The N-terminal sequence begins with VVGG, a motif commonly observed in mature snake-venom serine proteases, and the sequence identities reported for VLβF, calobin, and contortrixobin are substantially greater than that with human α-thrombin. Together with inhibition by aprotinin and partial inhibition by PMSF, these findings support classification of TLSP within the snake-venom serine-protease family [13].

The marked difference in apparent molecular mass between reducing and non-reducing SDS-PAGE conditions should be interpreted cautiously. Because no native molecular-mass or oligomeric-state analysis was performed, the observed migration difference cannot be attributed specifically to subunit dissociation or proteolytic processing and may instead reflect disulfide-dependent conformational effects or anomalous electrophoretic migration.

TLSP should nevertheless not be interpreted as a functional equivalent of thrombin. Although Boc-VPR-*pNA* was its preferred chromogenic substrate, TLSP showed much lower relative activity toward another thrombin-directed substrate, S-2238, and displayed substantial activity toward Boc-LGR-*pNA* and S-2288. The substantial activity toward several non-thrombin-directed chromogenic substrates indicates that TLSP has relatively broad active-site tolerance toward small synthetic substrates. Therefore, preference for Boc-VPR-*pNA* should not be interpreted as evidence of strict thrombin-like specificity. More importantly, TLSP preferentially degraded the fibrinogen β-chain and failed to induce fibrin-polymer formation. This phenotype resembles β-fibrinogenolytic venom serine proteases such as halystase and contortrixobin [13] and illustrates why chromogenic-substrate preference alone is insufficient to establish physiological thrombin-like function [8,14,15].

The cleavage of plasminogen and prothrombin after prolonged incubation should be interpreted cautiously. These experiments demonstrate proteolysis under the conditions tested, but do not establish physiological activation, cleavage-site specificity, or kinetic relevance. The TLSP-generated prothrombin fragments lacked thrombin activity, and TLSP did not degrade preformed or factor XIIIa-cross-linked fibrin. Consequently, the available experiments support proteolysis of these substrates but do not support prothrombin activation or plasmin-like fibrinolysis [3,16,17].

Thrombin is a multifunctional serine protease involved in hemostasis and other cellular responses [18]. Although coagulation-associated proteolysis can intersect with growth-factor signaling, including thrombin-mediated pro-HGFA activation [19,20,21], these pathways were not investigated in the present study.

A particularly notable feature of TLSP was its resistance to heat. Approximately 59% of amidolytic activity remained after exposure to 100 °C, and heat-treated enzyme retained β-fibrinogenolytic activity. TLSP was also PAS-positive and showed a molecular-mass shift after N-glycosidase F treatment. Previous studies of venom fibrinogenases have suggested that covalently attached carbohydrate can influence stability and enzyme properties [22,23]. N-glycosidase F treatment was accompanied by loss of detectable amidolytic activity, but the available data do not establish the underlying mechanism [24]. Importantly, the completeness of deglycosylation was not independently verified beyond the observed electrophoretic mobility shift [24]. Additional experiments would be required to determine whether N-linked glycans influence folding, active-site accessibility, solubility, stability, or other structural properties of TLSP [25].

The marked reduction in TLSP activity by β-mercaptoethanol and DTT suggests that disulfide bonds may be important for maintaining the catalytically active conformation of TLSP. However, because these reducing agents were tested at a relatively high concentration (500 mM), the present data do not establish a direct causal relationship between disulfide bonds and TLSP thermostability. Further structural studies are required to determine whether disulfide-bond stabilization contributes to its marked thermal resistance.

### Limitations and Future Directions

The principal limitation of the present dataset is that molecular identification is based on a short N-terminal sequence rather than a complete protein sequence, peptide mass fingerprint, LC-MS/MS profile, or corresponding gene sequence. The exact molecular identity of TLSP therefore remains unresolved. Accordingly, the present study should be interpreted as a biochemical characterization of the purified TLSP preparation rather than as the establishment of a novel molecular entity. In addition, although most quantitative assays were performed in duplicate and kinetic measurements were performed in three independent experiments, the original source records do not consistently provide measures of variation or inferential statistical analyses for all quantitative assays. Future work should confirm TLSP identity by contemporary mass-spectrometric or sequence-based methods, repeat key quantitative assays with predefined biological and technical replicates, and directly test the contribution of glycosylation to folding and thermal stability. These steps would strengthen structure–function interpretation while preserving the distinctive biochemical phenotype documented here. 

Although preferential cleavage of the fibrinogen β-chain was observed by SDS-PAGE, reliable densitometric quantification was limited by overlapping electrophoretic bands. Therefore, the relative degradation of the α- and β-chains could not be quantitatively determined.

The thermal-stability analysis was based on residual enzyme activity after a 30-min pre-incubation and did not assess reversibility of heat-induced changes, aggregation or precipitation, or complete thermal-inactivation kinetics. Therefore, the present data demonstrate marked heat resistance under the tested conditions but do not define its structural or thermodynamic basis.

We also did not determine whether TLSP-treated fibrinogen retains the ability to form fibrin following subsequent thrombin addition. This should be addressed in future studies to further define the functional consequences of TLSP-mediated fibrinogenolysis.

Future studies may examine whether TLSP can process plasminogen-related growth factors or their precursors, including HGF/HGFA- or MSP-related substrates, and whether such proteolysis influences downstream cellular responses [21,26,27,28]. These possibilities were not examined in the present study and remain to be investigated.

## 5. Conclusions

TLSP purified from *Gloydius ussuriensis* venom is a glycosylated serine protease that preferentially cleaves the fibrinogen β-chain and shows a preference for Boc-VPR-*pNA* among the chromogenic substrates tested, but does not display thrombin-like fibrin-clotting activity. The enzyme also showed marked heat resistance under the experimental conditions and did not degrade preformed or factor XIIIa-cross-linked fibrin. TLSP is operationally defined by its purification characteristics and N-terminal amino acid sequence. However, the exact molecular identity of TLSP remains unresolved, and the possibility that it corresponds to a previously described venom serine protease, such as gussorbin, cannot be excluded. Furthermore, the structural basis of its glycosylation- and heat-related properties remains to be elucidated. These data define the biochemical phenotype of the purified TLSP preparation and provide a basis for future molecular identification and structure–function studies.

## Figures and Tables

**Table 1:** **Purification summary of TLSP**.

Purification Step	Total Protein (mg)	Total Activity (U)	Specific Activity (U/mg)	Yield (%)
Crude venom	103.26	6195	60	100
HiPrep 16/10 Q FF	17.6	1584	90	25.5
Source 15 Q 4.6/100 PE	4.06	609	150	9.8
Superdex 75 10/300 GL	0.98	196	200	3.2
Mono Q HR 5/5 (1)	0.39	192	492	3.1
Mono Q HR 5/5 (2)	0.14	140	1000	2.3

One unit was defined in the original source study as the amount of enzyme hydrolyzing 0.25 mmol of Boc-VPR-*pNA* in 15 min at 37 °C.

**Table 2:** **Kinetic parameters of TLSP toward Boc-VPR-*pNA***.

Parameter	Value
*K*m	0.3 mM
*k*cat	8.69 s^−1^
*k*cat/*K*m	28.8 mM^−1^ s^−1^

**Table 3:** **N-terminal sequence comparison reported for TLSP**.

Protein/Source	N-Terminal Sequence	Identity (%)
TLSP, *Gloydius ussuriensis*	VVGGDEHNIN	100
VLβF, *Vipera lebetina*	VVGGDECNIN	90
Calobin, *Agkistrodon caliginosus*	VIGGDECNIN	80
Contortrixobin, *Agkistrodon contortrix contortrix*	VVGGDECNIN	90
Human α-thrombin	IVEGSDAEIG	30

**Table 4:** **Chromogenic-substrate specificity of TLSP**.

Chromogenic Substrate	Commonly Used Target Protease	Relative Activity (%)
Boc-VPR-*pNA*	Thrombin	100
Boc-LGR-*pNA*	Factor Xa	85
S-2222	Factor Xa	14
S-2238	Thrombin	27
S-2251	Plasmin	16
S-2288	t-PA/broad serine protease	81
S-2765	Factor Xa	28
S-2444	Urokinase	40

Activity was expressed relative to Boc-VPR-*pNA* (100%).

**Table 5:** **Effects of various inhibitors and reducing agents on TLSP enzyme activity**.

Additive	Relative Activity (%)
Control	100
EGTA	98.8
PMSF	61.4
β-Mercaptoethanol	31.6
DTT	15.5
Aprotinin	0.6
Bestatin	104.1
1,10-Phenanthroline	108.3
EDTA	96.3
TPCK	109.0
TLCK	82.8
DFP	99.5

EGTA, PMSF, aprotinin, bestatin, 1,10-phenanthroline, EDTA, TPCK, TLCK, and DFP were tested at 1 mM. β-Mercaptoethanol and DTT were tested at 500 mM. Enzyme activity was expressed relative to the untreated control (100%).

**Table 6:** **Effects of divalent cations on TLSP enzyme activity**.

Additive	Relative Activity (%)
Control	100
NiCl_2_	9.1
ZnCl_2_	1.4
MgCl_2_	99.3
MnCl_2_	98.5
CuCl_2_	17.5
CaCl_2_	97.2

Each divalent cation was tested at a final concentration of 1 mM. Enzyme activity was expressed relative to the untreated control (100%).

## Data Availability

The data presented in this study are available on request from the corresponding author.

## Supplementary Materials

The following supporting information can be downloaded at: https://www.mdpi.com/article/10.3390/biology15201892/s1, Figure S1: Original kinetic plot of TLSP toward Boc-VPR-pNA; Supplementary Materials File S1: Original images of the protein gels used in this study.

## Abbreviations

TLSP, thermostable serine protease; SVTLE, snake-venom thrombin-like enzyme; PAS, periodic acid–Schiff; PMSF, phenylmethanesulfonyl fluoride; DFP, diisopropyl fluorophosphate; EDTA, ethylenediaminetetraacetic acid; EGTA, ethylene glycol-bis(2-aminoethylether)-N,N,N′,N′-tetraacetic acid; DTT, dithiothreitol; BSA, bovine serum albumin; t-PA, tissue-type plasminogen activator.
