# Supplementary material for: Biochemical Characterization of a Purified Glycosylated β-Fibrinogenolytic Serine Protease Preparation from Gloydius ussuriensis Venom
Source: Biology (Basel). 2026 Oct 20;15(20):1892. doi: 10.3390/biology15201892 (PMC13701387; doi:10.3390/biology15201892)
Supplement: Supplementary file 1 [file biology-15-01892-s001.zip › biology-4610779-Supplementary figure S1.pdf]

Supplementary figure S1

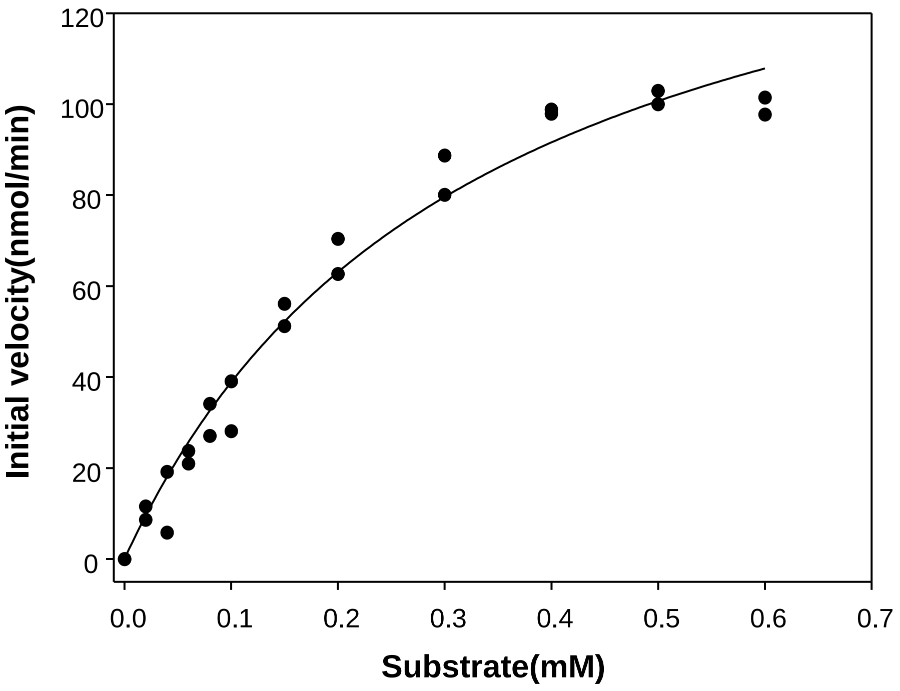

Figure S1. Original kinetic plot of TLSP toward Boc-VPR-*pNA*.The kinetic plot was retrieved from the original thesis presentation materials dated 28 May 2009. The assay was performed using 0.5 µg TLSP with Boc-VPR-pNA concentrations ranging from 0 to 0.6 mM at 37 °C. The originally reported kinetic parameters were Km = 0.3 mM, kcat = 8.69 s^{-1}, and kcat/Km = 28.8 mM^{-1} s^{-1}. The underlying numerical initial-velocity data were unavailable for independent reanalysis.
