# Supplementary material for: Biochemical Characterization of a Purified Glycosylated β-Fibrinogenolytic Serine Protease Preparation from Gloydius ussuriensis Venom
Source: Biology (Basel). 2026 Oct 20;15(20):1892. doi: 10.3390/biology15201892 (PMC13701387; doi:10.3390/biology15201892)

Figure 1F

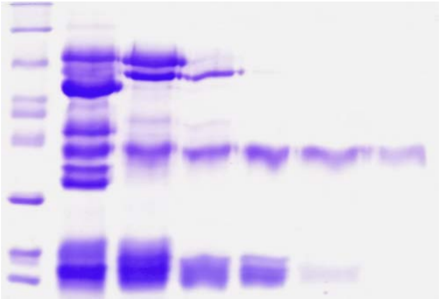

Figure 2A

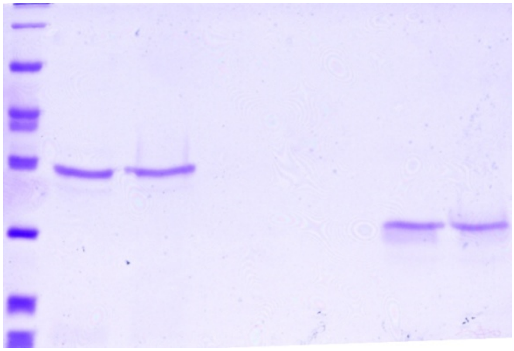

Figure 2B

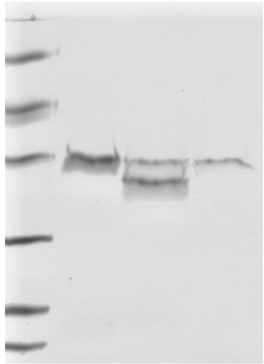

Figure 2C

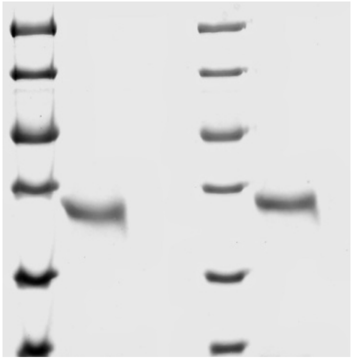

Figure 2D

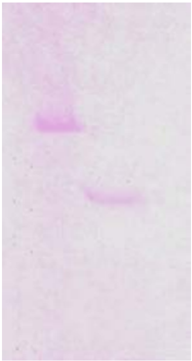

Figure 3A

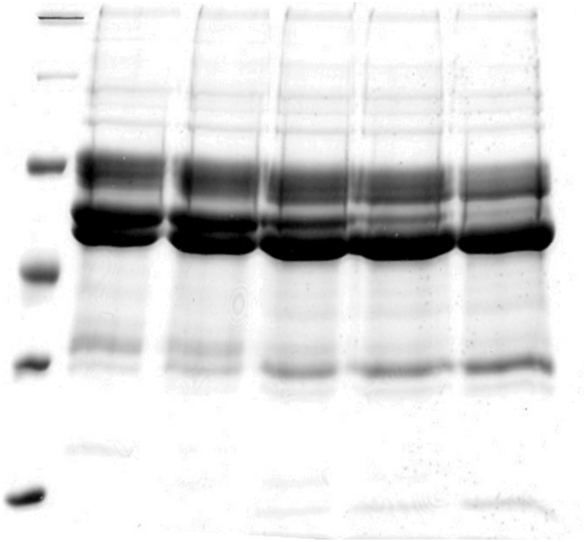

Figure 3B

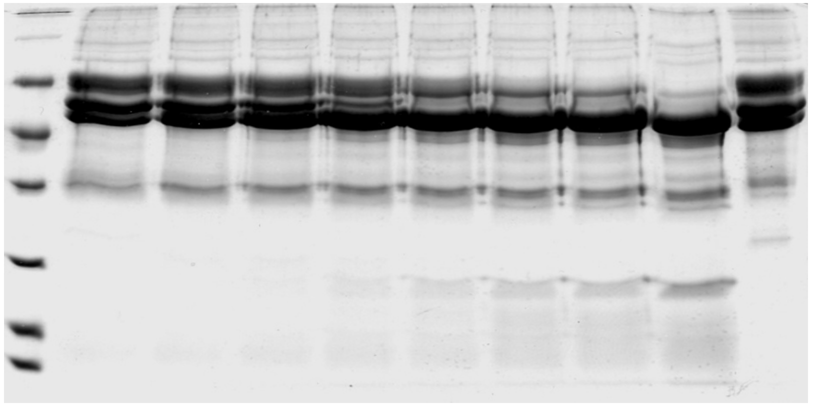

Figure 4B

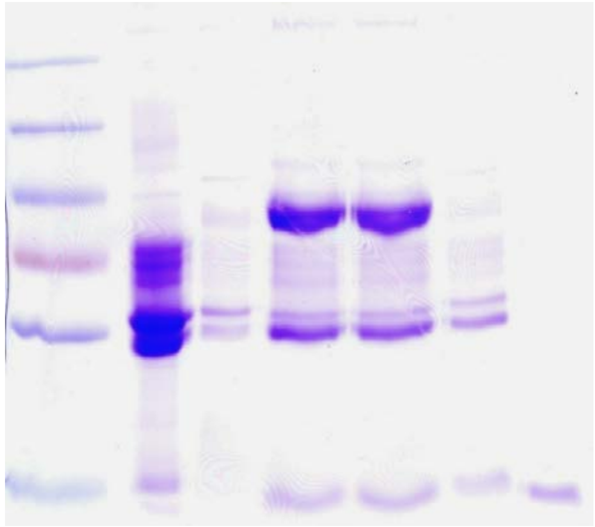

Figure 4C

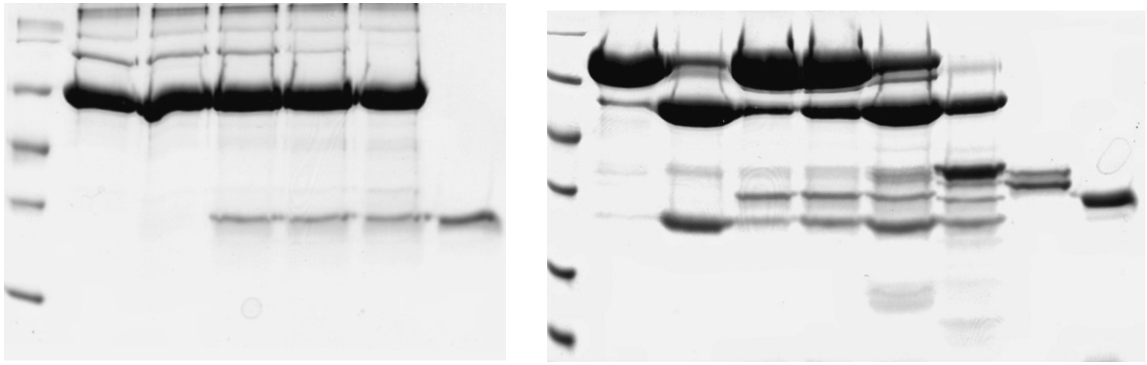

Figure 5C

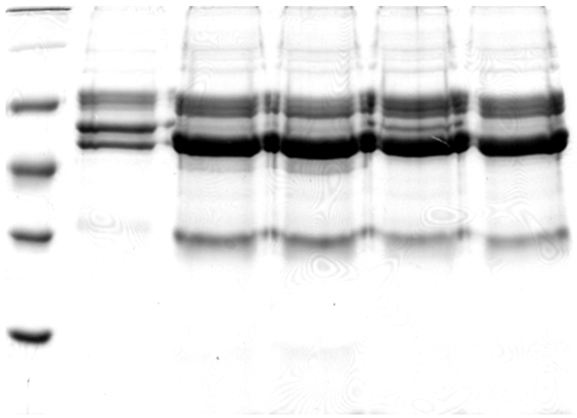

Supplement: Supplementary file 1 [file biology-15-01892-s001.zip › biology-4610779-Supplementary file S1.pdf]
